# The impacts of academic stress on college students' problematic smartphone use and Internet gaming disorder under the background of neijuan: Hierarchical regressions with mediational analysis on escape and coping motives

**DOI:** 10.3389/fpsyt.2022.1032700

**Published:** 2023-01-05

**Authors:** Xiao Gu, Eric (Zeqing) Mao

**Affiliations:** ^1^School of Marxism, Communication University of Zhejiang, Hangzhou, China; ^2^School of Cultural Creativity and Management, Communication University of Zhejiang, Hangzhou, China

**Keywords:** academic stress, problematic smartphone use, Internet gaming disorder, neijuan, escape, coping, China's education problems, behavioral addictions

## Abstract

With sluggish economic growth in the post-pandemic era, the phenomenon “neijuan” becomes increasingly severe in many Asian countries like China. Neijuan refers to a hypercompetitive social environment wherein individuals involuntarily get involved in inhumane work or study hours, resulting in a considerable amount of tension and stress. Previous pathology research has shown that stress can trigger the overuse of Internet-based devices and services, which can subsequently lead to problematic smartphone use (PSU) and Internet gaming disorder (IGD). Provided college students are generally deemed one of the groups most susceptible to neijuan, limited attention has been given to the stimuli and the resultant psychological and behavioral ill-beings. Our study examined the impacts of academic stress on Chinese college students' PSU and IGD problems, with the inclusion of escape and coping motives as mediators. Based upon the results of hierarchical regressions and path analysis, we found that whereas academic stress increased IGD tendency mediated through escape and coping motives, excessive use of smartphone might have developed into a habitual behavior rather than effective escape and coping instruments. Demographic and academic characteristics, such as gender and whether studying at a prestigious institution, also exerted influences on college students' IGD intensity.

## 1. Introduction

In addition to COVID-induced depression (1, 2), college students have to cope with substantial academic stress in the post-pandemic era, especially in Asian countries like China. As of 2022, there are 44.3 million students attending colleges and universities in China, not only making it home to the largest higher education system in the world (3) but also producing over 10 million graduates poised to enter the workforce (4). In view of this trend, academic performance deserves elevated importance mainly for two reasons. First and foremost, the pessimistic prospects about economic growth and grossly exacerbated difficulties in job search and employment have cultivated a prevailing fear of “neijuan,” which refers to a hypercompetitive social environment wherein individuals are forced to get involved in inhumane work or study hours, rather than creativity and innovation, as the sole means of maintaining and/or increasing their socioeconomic status (5). The phenomenon of neijuan highly resembles the “prisoner's dilemma” (6) in game theory, meaning someone's improvement in wellbeing occurs at the expense of others as opposed to additionally created wealth of the whole society. Notably, neijuan is most commonly observed among college students and high-tech employees in China (7). For many college students, graduate programs can be regarded as a temporary shelter, where they are allowed to wait for the economy to recover whilst gaining relevant skills and knowledge. In the face of relatively scarce opportunities of graduate admission, which are earned primarily by participating in a national test called *kaoyan*, today's Chinese college students have to put forth disproportionate effort to compete for the entry tickets, which can potentially intensify their susceptibility to academic stress. Second, on top of the rampant neijuan phenomenon, multiple incidents of academic plagiarism scandals in China's higher education institutions over the recent years have urged the administers to enact much more strict rules and policies on thesis and dissertation defenses (8), ending up with an enlarged number of student complaints about the increasingly demanding graduation requirements.

Previous pathology research has found that stress can readily trigger overuse of Internet-based devices and services (9–11). For example, although playing online games can be conducive to relieving stress, socializing, and enabling escapism from daily routines (12–14), uncontrolled and excessive engagement in Internet gaming tends to be closely aligned with maladaptive psychological and behavioral responses (15). In the education psychology literature, there is a growing body of research that reports a positive association between academic stress and Internet addiction, with particular interest in problematic smartphone use [PSU; (16–18)] and Internet gaming disorder [IGD; (19–21)]. Specifically, bearing striking resemblance to substance abuse, PSU entails withdrawal symptoms if unable to use the phone (22, 23), conflicts with family members or friends (24, 25), and relapses to addictive behaviors following a period of abstinence (26, 27). Aside from “problematic,” other terms such as “addictive,” “excessive,” “compulsive,” and “compensatory” have been used to portray PSU symptoms (28). On the other hand, while psychological and clinical studies had started to investigate IGD before smartphones became prevalent, PSU and IGD, having many similar negative outcomes such as those mentioned above, are increasingly examined and discussed in juxtaposition (29–32) considering smartphones' flexibility, portability, and accessibility, which greatly facilitate mobile play (28). Referred to as a “persistent and recurrent use of the Internet to engage in games, often with other players, leading to clinically significant impairment of distress” (p. 795), IGD was identified as a potential psychiatric disorder by the American Psychiatric Association (APA) and included in the third section of the fifth revision of the *Diagnostic and Statistical Manual of Mental Disorders* (DSM-5) (33). Moreover, in light of the growing social concerns surrounding pathological gaming, the World Health Organization (WHO) also incorporated “gaming disorder” in the *International Classification of Disorders*, 11th edition (ICD-11).

The compensatory Internet use (CIU) theory proposed by Kardefelt-Winther, (34, 35) conceptualizes addiction to the Internet as a means of compensating unsatisfied needs originated from the offline setting, which can help individuals cope with depression, anxiety, and stress. Researchers adopting the CIU model to analyze Internet addiction argue that it is warranted to better disentangle the mechanisms mediating the relationship between risk factors (e.g., stress) and pathological use of the Internet (36–38). The relevant literature has extensively explored smartphone and video game users' motivations. Pertaining to stress reduction and relaxation, escape and coping are believed to be important driving forces of prolonged time spent on smartphone usage (39–41) and Internet gaming (13, 14, 42, 43). Specifically, whereas escapism refers to “unidirectional and potentially permanent movement from the physical to the more favorably perceived gaming environment” [(44), p. 3], coping motives reflect “persistently changing cognitive and behavioral efforts in order to manage specific external and/or internal demands that are seen as taxing or exceeding the resources of the person” [(45), p. 3], Prior inquiries into the relationship between academic stress and PSU and IGD were often focused on young adolescents (17–19, 46–48). Provided sluggish economic growth in the post-pandemic era, college students, who have to confront increased difficulties in job search and employment, have become one of the most vulnerable groups to academic stress caused by neijuan. However, little is known about the maladaptive psychological and behavioral effects of neijuan on college students. With this in mind, the present paper aimed to examine how neijuan-related academic stress impacts Chinese college students' PSU and IGD problems, using escape and coping motives as the mediators. Accordingly, we propose the following hypotheses:

H1a. *Escape motive will mediate the relationship between academic stress and PSU*.
H1b. *Escape motive will mediate the relationship between academic stress and IGD*.
H2a. *Coping motive will mediate the relationship between academic stress and PSU*.
H2b. *Coping motive will mediate the relationship between academic stress and IGD*.

## 2. Methods

### 2.1. Participants

We capitalized on WeChat, one of the most widely used instant messaging and social media apps in China, to recruit participants and collected survey responses using the Tencent Questionnaire (https://wj.qq.com/). Specifically, 54 volunteers were recruited from a university in East China to participate in a prior pilot study, and after no major concerns arose, they helped send the survey link *via* personal WeChat account to their acquaintances (e.g., former high school classmates) who were enrolled in college as well. While 602 responses were initially received, 22 of them (3.65%) were dropped because of invalid answers (e.g., “1” or “male” for age; *N* = 7) or the participants not playing any video games at all (*N* = 15). Therefore, 580 valid responses were finally used for analysis. Among the respondents, 259 (45%) were male and 321 (55%) were female, and their ages ranged from 17 to 25 years, with a mean of 19.77 (SD = 1.30). In particular, 12% of the respondents were studying at higher education institutions listed in the so-called “985 and 211 project,” which are typically considered prestigious institutions in China [see (49)]. Descriptive statistics of more detailed academic information, including which year of college they were and their GPA rankings, appear in Table 1. We compared both the demographic and academic characteristics between male and female participants and found no significant gender differences in those characteristics. Our study was approved by the Institutional Review Board (IRB) of the university, and voluntary informed consent was obtained from the respondents, who were guaranteed anonymity and confidentiality.

**Table 1:** Demographic and academic characteristics grouped by gender.

**Variable**	**Male (*****N*** = **259)**		**Female (*****N*** = **321)**		
	**Mean or N**	**SD or %**	**Mean or** ***N***	**SD or %**	***t*** **or** **χ**^**2**^
Age	19.78	1.15	19.63	1.23	1.59
985 and 211 project	33	12.74%	37	11.53%	0.44
**Grade** ^a^					2.25
Freshman	44	16.99%	53	16.51%	
Sophomore	123	47.49%	184	57.32%	
Junior	59	22.78%	64	19.94%	
Senior	33	12.74%	20	6.23%	
**GPA ranking** ^a^					1.07
Top 25%	77	29.73%	117	36.45%	
25–50%	105	40.54%	125	38.94%	
50–75%	56	21.62%	54	16.82%	
75–100%	21	8.11%	25	7.79%	

*N* = 580.

^a^Fisher's exact tests, all *p* > 0.05.

### 2.2. Measurement

#### 2.2.1. Awareness of neijuan

We used two items to measure the participants' awareness of neijuan. For perceived severity, the respondents were asked to rate the item “Neijuan is common and severe in today's society” on a 5-point Likert scale (1 = *Strongly disagree*, 5 = *Strongly agree*). For concerns, the respondents scored the item “I am concerned about neijuan” also on a 5-point Likert scale (1 = *Strongly disagree*, 5 = *Strongly agree*).

#### 2.2.2. Academic stress

Items for measuring academic stress were adopted from Kohn and Frazer's (50) Academic Stress Scale (ASS), which has already been validated among Chinese college students (51). While the original ASS scale consisted of 35 items, the participants of the pilot study suggested that certain items might be less appropriate or relevant in the contemporary higher education environment of China. For example, students can use their laptops, tablets, or even smartphones to take notes if they do not have pencil/pen with them in class (i.e., the “forgetting pencil/pen” item). In light of this, we conducted exploratory factor analysis (EFA) to determine which items of the original ASS scale can be dropped so as to make a concise survey. Firstly, principal components analysis (PCA) was implemented to extract 35 factors. Second, eight components that have eigenvalues >1 were selected (52). Next, with factor loadings obtained from rerunning PCA using the eight components, we adopted a cutoff value of 0.4 (53) to decide which items to retain. Finally, out of the 35 items of the original ASS scale, 15 were eliminated and 20 were kept. The survey respondents were asked to evaluate the level of stress for academic stressors such as “Final grades,” “Excessive homework,” and “Class speaking” on a 5-point Likert scale (1 = *Not stressful*, 5 = *Extremely stressful*). Provided the removal of those items, the Cronbach's α still achieved 0.93, which suggested excellent internal consistency.

#### 2.2.3. Escape and coping motives

To evaluate college students' escape and coping motives, we used two subscales (four items for escape and four for coping) extracted from the Motives for online Gaming Questionnaire (MOGQ) developed by Demetrovics et al. (54). The Chinese version of the MOGQ scale, validated by Wu et al. (55), was applied in our study. The scale was slightly modified by incorporating “smartphone” into the descriptions. Sample items included “playing smartphone games helps me to forget about daily hassles” (escape) and “playing smartphone games helps me get into a better mood” (coping), with a 5-point Likert scale ranging from 1 (*Almost never*) to 5 (*Almost always*). As the Cronbach's αs for escape and coping motives were both 0.92, the items can be considered internally consistent.

#### 2.2.4. PSU

The respondents' PSU tendency was assessed with the Problematic Use of Mobile Phones (PUMP) scale developed by Merlo et al. (56). The scale contains 22 items with a 5-Likert scale (1 = *Strongly disagree*, 5 = *Strongly agree*). Sample items were “When I decrease the amount of time spent using my cell phone, I feel less satisfied” and “When I stop using my cell phone, I get moody and irritable.” The Cronbach's α being 0.95 indicated that the scale was internally consistent.

#### 2.2.5. IGD

In accordance with the DSM-5's essential criteria for IGD, Pontes and Griffiths (57) developed and validated a 9-item unidimensional scale, termed as IGDS9-SF, for evaluating and diagnosing IGD-related symptoms. The respondents scored their Internet gaming behavior based on a 5-point Likert scale ranging from 1 (*Never*) to 5 (*Very often*) for items such as “Do you feel more irritability, anxiety or even sadness when you try to either reduce or stop your gaming activity” and “Do you systematically fail when trying to control or cease your gaming activity.” Our study employed the simplified Chinese version of IGDS9-SF (58). The Cronbach's α was 0.96, meaning that the scale was internally consistent.

### 2.3. Statistical analysis

Prior to conducting regression analysis, we first computed the descriptive statistics for all measures. In particular, *t* or *F* tests were implemented to check for any significant differences in the variables of interest depending on the demographic and academic characteristics of the respondents. Then, two sets of hierarchical multiple regressions were conducted while designating psychological motivations (i.e., escape and coping) and behavioral outcomes (i.e., PSU and IGD) as the dependent variable, respectively. At Step 1, demographic and academic variables, including age, gender, 985 and 211 project, and GPA ranking, were used in the model specification. At Step 2, we took into account the respondents' awareness of neijuan, including their perceived severity of and concern for the increasingly competitive atmosphere. At Step 3, we then incorporated academic stress as well as escape and coping motives, if necessary, to estimate their impacts on the dependent variable under consideration. Based upon the results derived from the above steps, structural equation modeling (SEM) was utilized to conduct path analysis with the package lavaan (59) developed specifically for latent variable analysis in the software R. To evaluate model fitness, we adopted three indices, namely comparative fit index (CFI), incremental fit index (IFI), and root-mean squared error of approximation (RMSEA). With regard to the cut-offs of the fit indices, we considered values larger than 0.95 for CFI (60) and IFI (61) and values <0.05 for RMSEA (62) as signs of good model fit. Lastly, the significances of the direct and indirect effects were examined by constructing bias-corrected bootstrap confidence intervals based on 5,000 bootstrapped samples.

## 3. Results

### 3.1. Preliminary analysis

Table 2 displays the means and standard deviations of the variables analyzed in this study. Particularly, *t* or *F* statistic was provided to see if there existed any significant differences in the psychological and behavioral measures of interest, grouped by different demographic and academic conditions. According to the results of preliminary analysis, male college students tended to have higher scores on escape and coping motives than their female counterparts (*t* = 3.24, *p* < 0.01; *t* = 4.05, *p* < 0.001). Whereas the two genders barely differed in PSU (*t* = −0.09, *p* > 0.05), male participants were found to be more susceptible to IGD problems than females (*t* = 3.23, *p* < 0.01), which is consistent with previous findings in the literature (63–65). Interestingly, college students who were studying at prestigious institutions (i.e., 985 and 211 project) showed lower scores on escape and coping motives than those who were not (*t* = 3.49, *p* < 0.001; *t* = 4.90, *p* < 0.001). They were also less prone to IGD problems (*t* = 3.04, *p* < 0.01), though they did not display much difference in PSU tendency (*t* = 0.63, *p* > 0.05). Moreover, it is worth noting that the respondents' evaluations were not sensitive to which year they were studying in college and their GPA rankings (*p* > 0.05). Considering college year should be positively related with the respondent's age, it will not be included as a control variable in the following analysis. Table 3 reports the correlation matrix of the variables analyzed in this study.

**Table 2:** Descriptive statistics of psychological and behavioral variables analyzed in this study.

**Variable**	**Escape motive**		**Coping motive**		**PSU**		**IGD**		
	**Mean (SD)**	***t*** **/** ***F***	**Mean (SD)**	***t*** **/** ***F***	**Mean (SD)**	***t*** **/** ***F***	**Mean (SD)**	***t*** **/** ***F***
**Gender**		3.24^**^		4.05^***^		−0.09		3.23^**^
Male	2.36 (1.09)		2.83 (1.12)		2.92 (0.87)		2.04 (1.05)	
Female	2.06 (1.12)		2.44 (1.21)		2.92 (0.80)		1.77 (0.96)	
**985 and 211 project**		3.49^***^		4.90^***^		0.63		3.04^**^
Yes	1.77 (1.07)		2.01 (1.10)		2.85 (0.94)		1.56 (0.98)	
No	2.25 (1.11)		2.70 (1.17)		2.93 (0.81)		1.94 (1.00)	
**Grade**		0.49		0.28		0.31		0.13
Freshman	2.22 (1.06)		2.62 (1.06)		2.95 (0.96)		2.02 (1.00)	
Sophomore	2.14 (1.09)		2.59 (1.19)		2.90 (0.79)		1.83 (1.00)	
Junior	2.36 (1.19)		2.71 (1.23)		2.99 (0.80)		1.97 (1.08)	
Senior	1.83 (1.03)		2.22 (1.17)		2.83 (0.93)		1.70 (0.85)	
**GPA ranking**		2.14		0.71		1.55		0.79
Top 25%	2.23 (1.08)		2.62 (1.16)		3.06 (0.74)		1.81 (0.95)	
25–50%	2.19 (1.14)		2.62 (1.19)		2.94 (0.89)		1.92 (0.98)	
50–75%	2.35 (1.16)		2.76 (1.21)		2.89 (0.79)		2.04 (1.12)	
75–100%	1.68 (0.94)		2.20 (1.11)		3.13 (0.95)		1.75 (1.08)	

*N* = 580.

^*^p < 0.05,

^**^p < 0.01,

^***^p < 0.001.

**Table 3:** The correlation matrix of the variables analyzed in this study.

**Variable**	**1**	**2**	**3**	**4**	**5**	**6**	**7**	**8**	**9**	**10**	**11**
1. Age	1										
2. Gender	−0.07	1									
3.985 and 211 project	0.10^*^	−0.02	1								
4. GPA ranking	0.06	−0.05	−0.02	1							
5. Severity of neijuan	−0.01	0.11^**^	−0.05	0.07	1						
6. Concerns for neijuan	0.04	−0.01	−0.12^**^	0.02	0.45^***^	1					
7. Academic stress	0.00	0.01	−0.07	0.11^**^	0.33^***^	0.26^***^	1				
8. Escape motives	0.03	−0.13^**^	−0.14^***^	−0.06	0.11^**^	0.45^***^	0.30^***^	1			
9. Coping motives	0.00	−0.16^***^	−0.19^***^	−0.04	0.09^*^	0.41^***^	0.27^***^	0.77^***^	1		
10. PSU	−0.03	0.00	−0.03	0.02	0.23^***^	0.28^***^	0.50^***^	0.06	0.04	1	
11. IGD	0.03	−0.13^**^	−0.12^**^	0.04	0.05	0.32^***^	0.33^***^	0.73^***^	0.58^***^	0.34^***^	1

*N* = 580.

^*^p < 0.05,

^**^p < 0.01,

^***^p < 0.001.

### 3.2. Examining contributory factors to escape and coping motives

Tables 4, 5 present the results of hierarchical regression analysis on the factors contributing to escape and coping motives, respectively. To be precise, at Step 1 (i.e., Model 1) demographic and academic characteristics accounted for 4% of the variance of escape motive. At Step 2 (i.e., Model 2), the inclusion of neijuan-related factors, namely perceived severity and concerns, resulted in the model explaining 23% of the variance of escape motive. At the final step (i.e., Model 3), with the incorporation of academic stress, the specified model further explained 38% of the variance in escape motive. Age and GPA ranking were not significant predictors throughout the models. Based on the results of the full model (i.e., Model 3), it can be seen that female participants, compared to males, had a lower escape motive (*B* = −0.26, *p* < 0.01, β = −0.12). In addition, college students enrolled at 985 and 211 project institutions reported lower scores on escape motive than those who were not (*B* = −0.28, *p* < 0.05, β = −0.08). In terms of neijuan-related factors, whereas awareness about the severity of neijuan was negatively associated with escape motive (*B* = −0.19, *p* < 0.001, β = −0.16), concerns for neijuan significantly increased this motive (*B* = 0.43, *p* < 0.001, β = 0.45). Finally, academic stress was detected to positively predict the participants' propensity to escape from daily routines (*B* = 0.34, *p* < 0.001, β = 0.24).

**Table 4:** Hierarchical regression analysis on escape motives.

**Independent variables**	**Model 1**			**Model 2**			**Model 3**		
	***B***	**SE**	**β**	***B***	**SE**	**β**	***B***	**SE**	**β**
**Step 1**									
Age	0.03	0.04	0.04	0.01	0.03	0.01	0.01	0.03	0.01
Gender	−0.31^***^	0.09	−0.14^***^	−0.27^**^	0.08	−0.12^**^	−0.26^**^	0.08	−0.12^**^
211&985 project	−0.51^***^	0.14	−0.15^***^	−0.31^*^	0.13	−0.09^*^	−0.28^*^	0.12	−0.08^*^
GPA ranking	−0.09	0.05	−0.07	−0.09	0.04	−0.07	−0.11	0.06	−0.09
**Step 2**									
Severity of nejiuan				−0.11^*^	0.05	−0.10^*^	−0.19^***^	0.05	−0.16^***^
Concerns for neijuan				0.46^***^	0.04	0.48^***^	0.43^***^	0.04	0.45^***^
**Step 3**									
Academic stress							0.34^***^	0.05	0.24^***^
Adjusted *R*^2^	0.04			0.23			0.38		
*F* (4, 575)	6.68^***^			30.38^***^			33.35^***^		

*N* = 580.

^*^p < 0.05,

^**^p < 0.01,

^***^p < 0.001.

**Table 5:** Hierarchical regression analysis on coping motives.

**Independent variables**	**Model 1**			**Model 2**			**Model 3**		
	***B***	**SE**	**β**	***B***	**SE**	**β**	***B***	**SE**	**β**
**Step 1**									
Age	0.01	0.04	0.01	−0.01	0.04	−0.01	−0.01	0.04	−0.01
Gender	−0.40^***^	0.10	−0.17^***^	−0.37^***^	0.09	−0.16^***^	−0.36^***^	0.09	−0.15^***^
211&985 project	−0.71^***^	0.15	−0.20^***^	−0.52^***^	0.14	−0.14^***^	−0.49^***^	0.13	−0.14^***^
GPA ranking	−0.06	0.05	−0.05	−0.06	0.05	−0.05	−0.09	0.05	−0.07
**Step 2**									
Severity of nejiuan				−0.12^***^	0.05	−0.09^***^	−0.19^***^	0.05	−0.15^***^
Concerns for neijuan				0.43^***^	0.04	0.43^***^	0.41^***^	0.04	0.40^***^
**Step 3**									
Academic stress							0.33^***^	0.06	0.22^***^
Adjusted *R*^2^	0.06			0.21			0.32		
*F*(4, 575)	10.32^***^			27.20^***^			29.08^***^		

*N* = 580.

^*^p < 0.05,

^**^p < 0.01,

^***^p < 0.001.

Regarding coping motive, demographic and academic characteristics (Mode 1) explained 6% of its variance. In Model 2, the integration of perceived severity of and concerns for neijuan collectively contributed to the model accounting for 21% of the variance. In Model 3, by incorporating academic stress, the model rationalized 32% of the variance in coping motive. Similar to the case of escapism, age and GPA ranking were not significant predictors for coping motive throughout the models. According to the results of the full model (Model 3), female participants tended to score lower on coping motive than their male counterparts (*B* = −0.36, *p* < 0.001, β = −0.15). Also, 985 and 211 project college students were found to have a lower coping motive than those from non-prestigious schools (*B* = −0.49, *p* < 0.001, β = −0.14). For neijuan-related factors, perceived severity of neijuan attenuated coping motive (*B* = −0.19, *p* < 0.001, β = −0.15) while concerns about neijuan intensified this motive (*B* = 0.41, *p* < 0.001, β = 0.40). Lastly, academic stress generated a significantly positive effect on the participants' inclination to cope with unintended emotions (*B* = 0.33, *p* < 0.001, β = 0.22).

### 3.3. Examining contributory factors to PSU and IGD

Tables 6, 7 present the results of hierarchical regression analysis on the variables causing PSU and IGD symptoms, respectively. For PSU, the respondents' demographic and academic characteristics explained only 1% of the variance in Model 1. The inclusion of neijuan-related factors led to a model (Model 2) explaining 10% of the variance of PSU problems. In Model 3, the addition of academic stress as well as escape and coping motives accounted for 32% of the variance. Interestingly, in the full model (Model 3), all of the demographic and academic characteristics as well as neijuan-related factors were not significant predictors. However, academic stress significantly increased the respondents' PSU propensity (*B* = 0.41, *p* < 0.001, β = 0.39) and so did escape and coping motives (*B* = 0.19, *p* < 0.001, β = 0.26; *B* = 0.12, *p* < 0.001, β = 0.14).

**Table 6:** Hierarchical regression analysis on problematic smartphone use.

**Independent variables**	**Model 1**			**Model 2**			**Model 3**		
	***B***	**SE**	**β**	***B***	**SE**	**β**	***B***	**SE**	**β**
**Step 1**									
Age	−0.02	0.03	−0.03	−0.03	0.03	−0.05	−0.03	0.02	−0.05
Gender	0.01	0.07	0.01	−0.01	0.07	−0.01	0.06	0.06	0.04
211&985 project	−0.06	0.11	−0.02	0.03	0.10	0.01	0.13	0.09	0.05
GPA ranking	0.11	0.09	0.12	0.10	0.08	0.11	0.09	0.08	0.09
**Step 2**									
Severity of nejiuan				0.11^**^	0.04	0.12^**^	0.04	0.04	0.05
Concerns for neijuan				0.16^***^	0.04	0.22^***^	0.03	0.03	0.04
**Step 3**									
Academic stress							0.41^***^	0.04	0.39^***^
Escape motives							0.19^***^	0.04	0.26^***^
Coping motives							0.12^***^	0.02	0.14^***^
Adjusted *R*^2^	0.01			0.10			0.32		
*F* (4, 575)	5.85^***^			11.23^***^			31.81^***^		

*N* = 580.

^*^p < 0.05,

^**^p < 0.01,

^***^p < 0.001.

**Table 7:** Hierarchical regression analysis on Internet gaming disorder.

**Independent variables**	**Model 1**			**Model 2**			**Model 3**		
	***B***	**SE**	**β**	***B***	**SE**	**β**	***B***	**SE**	**β**
**Step 1**									
Age	0.02	0.03	0.03	0.01	0.03	0.01	0.03	0.02	0.04
Gender	−0.27^**^	0.08	−0.13^**^	−0.24^**^	0.08	−0.12^**^	−0.18^**^	0.06	−0.16^**^
211&985 project	−0.40^**^	0.13	−0.13^**^	−0.27^*^	0.12	−0.09^*^	−0.06	0.09	−0.02
GPA ranking	0.03	0.05	0.03	0.03	0.04	0.03	0.06	0.07	0.06
**Step 2**									
Severity of nejiuan				−0.11	0.09	−0.10	−0.07	0.08	−0.07
Concerns for neijuan				0.30^***^	0.04	0.35^***^	0.24^***^	0.03	0.16^***^
**Step 3**									
Academic stress							0.18^***^	0.04	0.13^***^
Escape motives							0.62^***^	0.04	0.68^***^
Coping motives							0.22^***^	0.03	0.26^***^
Adjusted *R*^2^	0.03			0.13			0.55		
*F* (4, 575)	5.26^***^			14.89^***^			80.19^***^		

*N* = 580.

^*^p < 0.05,

^**^p < 0.01,

^***^p < 0.001.

For IGD, the respondents' demographic and academic characteristics explained 3% of the variance (Model 1). The inclusion of neijuan-related factors contributed to a model (Model 2) explaining 13% of the variance. By incorporating academic stress and escape and coping motives, the model (Model 3) finally accounted for 55% of the variance in IGD scores. According to the results of the full model (Model 3), whereas age and 985 and 211 project did not serve as significant predictors for IGD problems, female college students tended to be less susceptible to online gaming addiction (*B* = −0.18, *p* < 0.01, β = −0.16). Furthermore, although the respondents seemed not to be sensitive to the perceived severity of neijuan (*B* = −0.07, *p* > 0.05, β = −0.07), their concerns for neijuan significantly increased their IGD tendency (*B* = 0.24, *p* < 0.001, β = 0.16). Last but not least, academic stress was found to be an important external stimulus of IGD symptoms (*B* = 0.18, *p* < 0.001, β = 0.13), while both escape and coping motives functioned psychologically in increasing IGD tendency (*B* = 0.62, *p* < 0.001, β = 0.68; *B* = 0.22, *p* < 0.001, β = 0.26).

### 3.4. Mediational analyses

**Figure 1:**
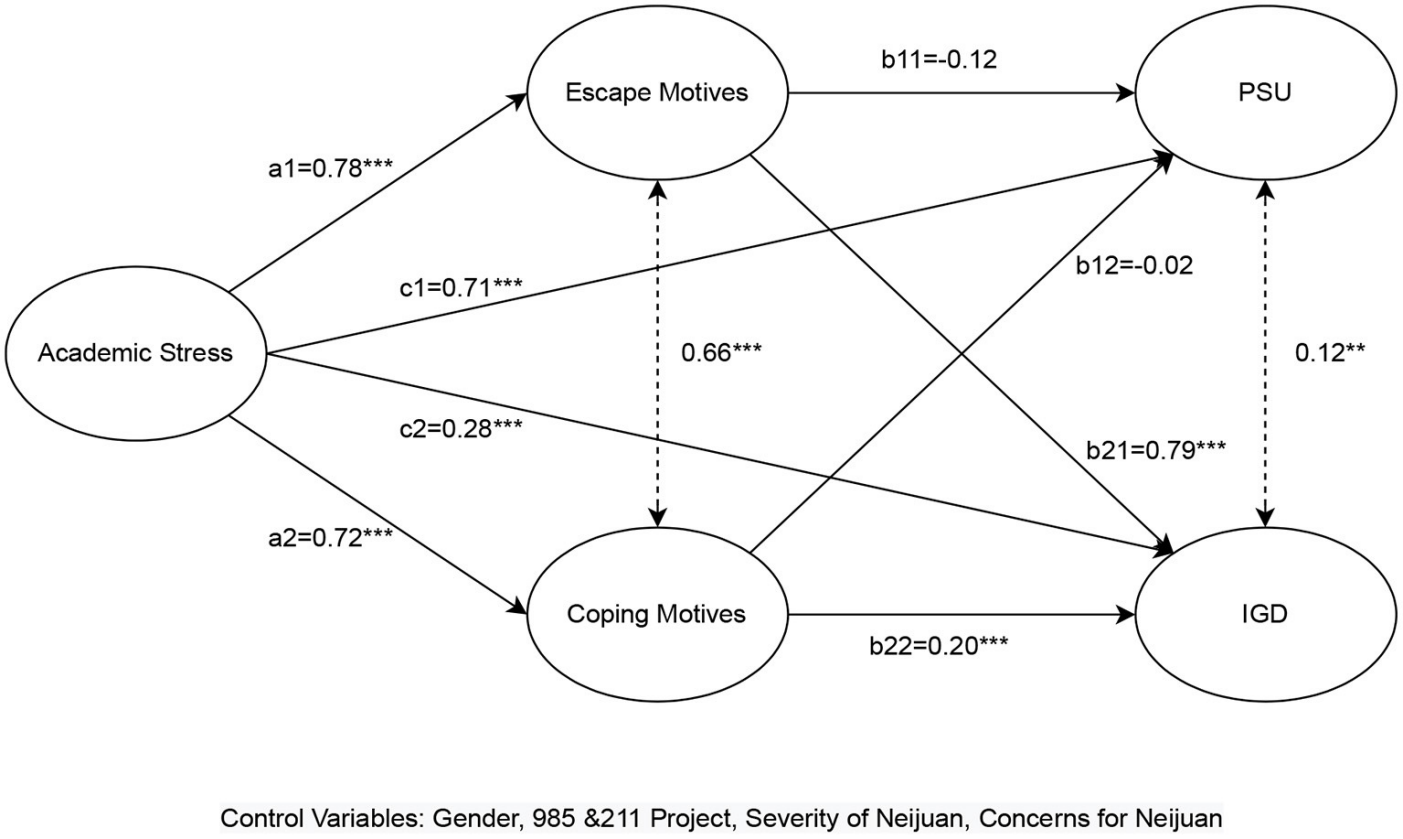
Results of mediational analysis. Path coefficients are unstandardized. *N* = 580. ^*^p < 0.05, ^**^p < 0.01, ^***^p < 0.001.

The results of mediational analyses are provided in Table 8 and Figure 1, with factors including gender, 985 and 211 project, perceived severity of neijuan, and concerns for neijuan being controlled for. Our findings revealed that, while academic stress had a direct positive effect on PSU (c1: *B* = 0.71, *p* < 0.001, β = 0.44), its indirect effects mediated *via* escape and coping motives were not significant (a1b11: *B* = −0.09, *p* > 0.05 β = −0.04; a1b12: *B* = −0.02, *p* > 0.05, β = −0.01). Nevertheless, the total effect of academic stress on PSU was still significant (*B* = 0.61, *p* < 0.001, β = 0.45). With regard to path analysis on IGD symptoms, in addition to a significantly positive direct effect (c2: *B* = 0.28, *p* < 0.001, β = 0.12), academic stress exerted indirect impacts on the respondents IGD tendency through escape and coping motives (a2b21: *B* = 0.57, *p* < 0.001, β = 0.25; a2b22: *B* = 0.15, *p* < 0.01, β = 0.10). The model fit indices were CFI = 0.98, TLI = 0.96, RMSEA = 0.04, pointing to good model fitness (66). In summary, the results of mediational analyses supported H1b and H2b but rejected H1a and H2a.

**Table 8:** Results of mediational analysis.

**Paths**	***B***	**SE**	**β**	**95% CI for *B***	**Hypothesis**
Academic stress -> escape motives (a1)	0.78^***^	0.13	0.32	[0.53, 1.02]	
Academic stress -> coping motives (a2)	0.72^***^	0.14	0.30	[0.44, 1.01]	
Escape motives -> PSU (b11)	−0.12	0.07	−0.13	[−0.26, 0.03]	
Coping motives -> PSU (b12)	−0.02	0.06	−0.04	[−0.14, 0.10]	
Escape motives -> IGD (b21)	0.79^***^	0.08	0.83	[0.64, 0.95]	
Coping motives -> IGD (b22)	0.20^***^	0.06	0.30	[0.08, 0.32]	
**Direct effect**					
Academic stress -> PSU (c1)	0.71^***^	0.11	0.44	[0.49, 0.94]	
Academic stress -> IGD (c2)	0.28^**^	0.10	0.12	[0.06, 0.48]	
**Indirect effect**					
Academic stress -> escape motives -> PSU (a1^*^b11)	−0.09	0.07	−0.04	[−0.22, 0.06]	H1a
Academic stress -> coping motives -> PSU (a1^*^b12)	−0.02	0.05	−0.01	[−0.12, 0.08]	H1b
Academic stress -> escape motives -> IGD (a2^*^b21)	0.57^***^	0.14	0.25	[0.30, 0.85]	H2a
Academic stress -> coping motives -> IGD (a2^*^b22)	0.15^**^	0.05	0.10	[0.05, 0.25]	H2b
**Total effect**					
PSU (c1+a1^*^b11+a1^*^b12)	0.61^***^	0.10	0.45	[0.42, 0.81]	
IGD (c2+a2^*^b21+a2^*^b22)	0.99^***^	0.14	0.65	[0.70, 1.26]	

*N* = 580.

^*^p < 0.05,

^**^p < 0.01,

^***^p < 0.001.

## 4. Discussion

Provided the growing awareness of and concerns for neijuan, especially in Asian countries like China, Japan, and South Korea, only limited academic attention has been given to this topic [e.g., (67, 68)]. The current study empirically examined the impacts of academic stress on Chinese college students, a group particularly susceptible to neijuan, with the focus on their escape and coping motives as well as their likelihood to suffer from PSU and IGD problems. The results in our study indicated that high academic stress was positively correlated with college students' motives to seek escapism from daily routines and adopt coping methods for alleviating negative status, which resonated with the broader discussions surrounding stress reduction (69–71). Academic stress was also closely aligned with college students' susceptibilities to PSU and IGD, which helped confirm those of previous research reporting positive associations between stress and PSU and IGD among college students (18, 72–74). Furthermore, escape and coping motives were found to serve as important underlying mediators that can help interpret the relation between academic stress and IGD tendency. It has been well established in the literature that the need to find an outlet for escape from reality and maladaptive coping styles can positively predict Internet addiction (75, 76), which is especially the case for playing video games as well as watching video game-themed contents (77–79). Radically different from IGD, there was no significant indirect association between college students' academic stress and PSU tendency through such a mediating mechanism. This finding implied that smartphone use might have already developed into a habitual behavior instead of effective escape and coping instruments.

Some other interesting findings were also obtained in our study. For example, the participants who expressed greater concerns for neijuan were inclined to have higher escape and coping motives as well as stronger PSU and IGD tendencies. In contrast, those who were fully conscious of neijuan did not give high scores on the above measures, implying they might have taken neijuan for granted. This can also be justified by Antonovsky's (80) sense of coherence, referring to comprehensibility as the extent to which people might cognitively perceive both internal and external stimuli as being understandable in some kinds of rational way. Notably, female participants in our study had significantly lower scores on escape and coping motives than their male counterparts, which is vastly different from prior investigations on substance and behavioral addictions (78, 81). In addition, whereas GPA ranking was not effective in predicting the participants' PSU and IGD motivations and behaviors, college students studying at prestigious higher education institutions tended to have lower escape and coping motives and were less prone to IGD problems than those who were not. Therefore, this finding suggested an inter-institutional, rather than intra-institutional, difference in the related risk factors among college students.

Our study had several limitations. First, other variables pertaining to the theory of behaviors in question were not examined. For example, previous research has found that poor interpersonal relationships can impose emotional stress on college students (82). Accordingly, it follows that social exclusion within the campus context may influence college students' PSU and IGD propensities. Second, the cross-sectional nature of the data in our study can only unveil the relationships between the variables of interest but does not necessarily ensure causal inferences. Therefore, future research might consider adopting longitudinal or experimental designs to infer causal relationships. Third, our sample was restricted to college students in China, which may compromise the generalizability of the findings due to underlying cultural differences.

## 5. Conclusion

Our study found that neijuan-related academic stress tremendously impacted college students' PSU and IGD motivations and symptoms. The findings suggested that, whereas academic stress increased IGD tendency mediated *via* escape and coping motives, overuse of smartphone might have developed into a habitual behavior as opposed to effective escape and coping tools. Moreover, demographic and academic characteristics, such as gender and whether studying at a prestigious institution, also exerted influences on college students' IGD problems.

## Data availability statement

The raw data supporting the conclusions of this article will be made available by the authors, without undue reservation.

## Ethics statement

The studies involving human participants were reviewed and approved by Institutional Review Board, School of Cultural Creativity and Management, Communication University of Zhejiang. The patients/participants provided their written informed consent to participate in this study.

## Author contributions

XG and EM contributed to conception and design of the study. EM organized the database, performed the statistical analysis, and wrote sections of the manuscript. XG wrote the first draft of the manuscript. All authors contributed to manuscript revision, read, and approved the submitted version.
